# Hepatitis B Virus Core Promoter Double Mutations (A1762T, G1764A) Are Associated with Lower Levels of Serum Dihydrolipoyl Dehydrogenase

**DOI:** 10.1159/000445319

**Published:** 2016-06-16

**Authors:** Zhi-Hua Jiang, Qin-Yan Chen, Tim J. Harrison, Guo-Jian Li, Xue-Yan Wang, Hai Li, Li-Ping Hu, Kai-Wen Li, Qing-Li Yang, Chao Tan, Zhong-Liao Fang

**Affiliations:** ^a^Guangxi Zhuang Autonomous Region Center for Disease Prevention and Control, Guangxi Key Laboratory for the Prevention and Control of Viral Hepatitis, Nanning, Guangxi, PR China; ^b^Division of Medicine, UCL Medical School, London, UK; ^c^Department of Public Health of Guangxi Zhuang Autonomous Region, Nanning, Guangxi, PR China; ^d^School of Preclinical Medicine, Guangxi Medical University, Nanning, Guangxi, PR China

**Keywords:** Hepatitis B virus, Basal core promoter, Mutations, Dihydrolipoyl dehydrogenase, Proteomics

## Abstract

**Objectives:**

The aim of this study was to identify serum proteins with differential concentrations between hepatocellular carcinoma (HCC) patients and HBsAg asymptomatic carriers among individuals infected with hepatitis B virus (HBV) with basal core promoter (BCP) double mutations (A1762T, G1764A).

**Methods:**

iTRAQ and liquid chromatography-tandem mass spectrometry were used to identify differentially expressed protein, and an ELISA test was used for the validation test.

**Results:**

The total number of proteins identified was 1,125, of which 239 showed statistically significant differences in their expression. The relative concentrations of serum dihydrolipoyl dehydrogenase (DLD), which showed the most significant correlation with liver diseases and infection, were significantly lower in HCC patients than asymptomatic HBsAg carriers and individuals negative for HBsAg. However, only the difference between HCC patients with BCP double mutations and HBsAg-negative individuals could be confirmed by ELISA. Meanwhile, we found that the concentrations of serum DLD in those infected with HBV with BCP double mutations were significantly lower than in individuals with the wild-type BCP. However, the difference in the concentrations of serum DLD between individuals with wild-type BCP and those negative for HBsAg was not significant.

**Conclusions:**

HBV with BCP double mutations are associated with lower concentrations of serum DLD.

## Introduction

Hepatocellular carcinoma (HCC) is the most common primary malignancy of the liver. Globally, it is the fifth most common cancer in men, and seventh among women, with over half a million new cases diagnosed annually worldwide [1]. The great majority of liver cancer (>80%) occurs in either sub-Saharan Africa or in Eastern Asia, with one country alone, China, accounting for over 50% of cases [2]. When specific diagnostic tests for hepatitis B virus (HBV) infection were introduced in the early 1970s, it became clear that there is a strong etiological association between chronic HBV infection and the development of HCC [3]. However, the mechanisms of oncogenesis remain obscure.

HBV has a circular, partially double-stranded DNA genome of about 3,200 nt with four open reading frames (ORFs), namely the core/precore, polymerase, surface and X ORFs [4]. Transcription of the four ORFs is controlled by the core, large surface, major surface and X promoters. The core promoter, located between nt 1,575 and 1,849, consists of the basal core promoter (BCP; nt 1,743-1,849) and the upper regulatory region (nt 1,613-1,742) [5]. The genome replicates via an RNA intermediate and the lack of proofreading during reverse transcription of the pregenomic RNA favors the development of sequence variants during long-term HBV replication [6]. One of the most critical changes is the appearance of double mutations at nt 1,762 (A-T) and 1,764 (G-A) in the BCP [7]. This has been reported to be associated with the pathogenesis of progressive liver disease, including HCC [8,9,10,11].

In our initial studies [9,10], we found BCP double mutations in HBV from 96% of HCC patients, but only 24% of controls, from Guangxi, China. In order to determine the value of screening HBsAg carriers for virus with BCP double mutations as a marker of extremely high risk of developing HCC, we are carrying out a prospective cohort study in Long An county, southern Guangxi. Within this cohort, we found that 93.3% of the male HCC cases were infected with the mutant [12]. Clearly, the double mutations are an etiological factor of HCC. The prevalence of BCP double mutations in the population where the cohort was established is 53%. It is also clear that not all of those infected by HBV with BCP double mutations ultimately develop HCC. What is the difference between those who will develop HCC and those who will not, among individuals infected with HBV with BCP double mutations? The answer will enable us to understand further the mechanisms of oncogenesis of HBV and, probably, obtain a good biomarker for HCC.

The aim of this study was to identify serum proteins with differential concentrations among HCC patients infected with HBV with BCP double mutations, asymptomatic HBsAg carriers with BCP double mutations and individuals negative for HBsAg, using iTRAQ (isobaric tags for relative and absolute quantification) [13] and liquid chromatography-tandem mass spectrometry.

## Materials and Methods

### Serum Sample Collection

Serum samples were collected from patients from Guangxi Medical University and the Long An cohort [12]. The patients who provided serum samples for proteomic analysis and for the validation of the significantly differentially expressed protein included HCC cases, asymptomatic HBsAg carriers and HBsAg-negative individuals. All blood samples were incubated at room temperature for 30 min and centrifuged for 10 min at 3,000 rpm (1,600 *g*). The serum samples were divided into three aliquots and processed independently as biological triplicates to assess the reproducibility of the data. The sera were stored at −80°C until the analysis.

Informed consent in writing was obtained from each individual. The study protocol conformed to the ethical guidelines of the 1975 Declaration of Helsinki and was approved by the Guangxi Institutional Review Board.

### Serological Testing and HBV DNA Amplification and Sequencing

The tests for HBsAg, HBeAg/anti-HBe and alanine aminotransferase (ALT) in serum, and amplification of the BCP of HBV DNA and nucleotide sequencing were as described previously [12].

### Protein Preparation

To reduce the complexity of the samples, highly abundant proteins were depleted using ProteoMiner™ kits (Bio-Rad Laboratories, Hercules, Calif., USA) according to the manufacturer's protocol. Briefly, the samples were eluted in Lysis buffer (7 M urea, 2 M thiourea, 4% CHAPS, 40 mM Tris-HCl, pH 8.5) and reduced with 10 mM DTT (final concentration) at 56°C for 1 h, followed by alkylation with 55 mM IAM (final concentration) for 1 h in the dark. The reduced and alkylated protein mixtures were precipitated by adding 4× volume of chilled acetone at −20°C overnight. After centrifugation at 4°C, 30,000 *g*, the pellet was dissolved in 0.5 M TEAB (Applied Biosystems, Milan, Italy) and sonicated on ice. After centrifuging at 30,000 *g* at 4°C, an aliquot of the supernatant was taken for the determination of protein concentration using the Bradford method with BSA as a standard [14]. The proteins in the supernatants were kept at −80°C for further analysis.

### iTRAQ Labeling and SCX Fractionation

Total protein (100 μg) was taken from each sample solution and digested with Trypsin Gold (Promega, Madison, Wis., USA), with a ratio of protein:trypsin of 30:1, for 16 h at 37°C. After trypsin digestion, the peptides were dried by vacuum centrifugation. The peptides were reconstituted in 0.5 M TEAB and processed according to the manufacturer's protocol for the 8-plex iTRAQ reagent (Applied Biosystems). Briefly, 1 unit of iTRAQ reagent was thawed and reconstituted in 24 μl of isopropanol. Peptides were labeled with 116, 118 and 121 iTRAQ tags by incubation at room temperature for 2 h. The labeled peptide mixtures were then pooled and dried by vacuum centrifugation.

SCX (strong cation exchange) chromatography was performed with an LC-20AB HPLC Pump system (Shimadzu, Kyoto, Japan). The iTRAQ-labeled peptide mixtures were reconstituted with 4 ml of buffer A (25 mM NaH_2_PO_4_ in 25% ACN, pH 2.7) and loaded onto 4.6 × 250 mm Ultremex SCX columns containing 5-µm particles (Phenomenex). The peptides were eluted at a flow rate of 1 ml/min with a gradient of buffer A for 10 min, 5-60% buffer B (25 mM NaH_2_PO_4_, 1 M KCl in 25% ACN, pH 2.7) for 27 min, and 60-100% buffer B for 1 min. The system was then maintained at 100% buffer B for 1 min before equilibrating with buffer A for 10 min prior to the next injection. Elution was monitored by measuring the absorbance at 214 nm and fractions were collected every 1 min. The eluted peptides were pooled into 20 fractions, desalted with a Strata X C18 column (Phenomenex) and vacuum dried.

### LC-ESI-MS/MS Analysis Based on Triple TOF 5600

LC-ESI-MS/MS (liquid chromatography-electrospray ionization tandem mass spectrometry) analysis based on Triple TOF (time-of-flight) 5,600 was performed. Each fraction was resuspended in buffer A (5% ACN, 0.1% FA) and centrifuged at 20,000 *g* for 10 min; the final concentration of peptide was about 0.5 µg/µl on average. Ten microliters of supernatant was loaded onto an LC-20AD nanoHPLC (Shimadzu, Kyoto, Japan) by the autosampler onto a 2-cm C18 trap column. The peptides were then eluted onto a 10-cm analytical C18 column (inner diameter 75 µm) packed in-house. The samples were loaded at 8 µl/min for 4 min, then the 35-min gradient was run at 300 nl/min starting from 2 to 35% B (95% ACN, 0.1% FA), followed by a 5-min linear gradient to 60%, then a 2-min linear gradient to 80%, maintenance at 80% B for 4 min, and finally a return to 5% for 1 min.

Data acquisition was performed with a Triple TOF 5600 system (AB SCIEX, Concord, Ont., Canada) fitted with a Nanospray III source (AB SCIEX) and a pulled quartz tip as the emitter (New Objectives, Woburn, Mass., USA). Data were acquired using an ion spray voltage of 2.5 kV, curtain gas of 30 psi, nebulizer gas of 15 psi, and an interface heater temperature of 150°C. The MS was operated with a reverse phase greater than or equal to 30,000 full width at half maximum for TOF MS scans. For IDA, survey scans were acquired in 250 ms and as many as 30 product ion scans were collected if exceeding a threshold of 120 counts/s and with a 2+ to 5+ charge state. The total cycle time was fixed to 3.3 s. The Q2 transmission window was 100 Da for 100%. Four time bins were summed for each scan at a pulser frequency value of 11 kHz through monitoring of the 40-GHz multichannel TDC detector with four-anode channel detection. A sweeping collision energy setting of 35 ± 5 eV coupled with iTRAQ adjust rolling collision energy was applied to all precursor ions for collision-induced dissociation. The dynamic exclusion was set for 1/2 of the peak width (15 s) and then the precursor was refreshed off the exclusion list.

### Data Analysis

Mass spectra from the 20 fractions were combined into one MGF (Mascot generic format) file, and the MGF file was searched against the International Protein Index (IPI) human sequence databases (version 3.87, human, 91,464 sequences) using Mascot software (version 2.3.02; Matrix Science, London, UK). The search parameters were as follows: the fixed modifications were carbamidomethyl (C), iTRAQ8plex (N-term) and iTRAQ8plex (K), and the variable modifications were Gln→pyro-Glu (N-term Q), oxidation (M) and iTRAQ8plex (Y). The peptide mass tolerance was 0.05 Da and fragment mass tolerance was 0.1 Da. Trypsin was chosen as the enzyme with one missed cleavage allowed; an automatic decoy database search strategy was employed to estimate the FDR (false discovery rate). In the final search results, the FDR was <0.05. All identified peptides had an ion score above the Mascot peptide identity threshold and a protein was considered identified if at least one such unique peptide match was apparent for the protein. For protein quantitation, the filters were set as follows: ‘median’ was chosen for the protein ratio type; the minimum precursor charge was set to 2+ and minimum peptide was set to 2; only unique peptides were used to quantify proteins. The median intensities were set as normalization and outliers were removed automatically. Proteins with a 1.2-fold or more change and p value <0.05 were considered as differentially expressed proteins. The logarithm of the ratio to base 2 below zero was considered to be downregulated, whereas those above zero were considered upregulated. Gene Ontology (GO) functional classifications were analyzed with Blast2GO software (http://www.blast2go.org/) and GO enrichment analysis was performed to identify GO terms that were significantly enriched in differentially expressed proteins.

### Validation of the Significantly Differentially Expressed Protein Dihydrolipoyl Dehydrogenase by ELISA

Dihydrolipoyl dehydrogenase (DLD) in sera was measured using an ELISA kit for DLD (Shanghai Xin Yu Biotech Co. Ltd, Shanghai, China) according to the manufacturer's instructions. The concentration of the protein in each sample was determined from a standard curve based on serial dilutions of the protein standard provided in the kit.

### Statistical Analysis

Results for continuous variables with normal distributions are given as the mean ± standard deviation (SD). Student's t test was used to compare means between two groups. The results for continuous variables that were not normally distributed are given as the median (range) and were compared using the Mann-Whitney U test. Multiple linear regression analyses were performed to identify independent factors associated with concentrations of proteins identified in serum. If necessary, a logarithmic transformation was applied to concentrations of identified proteins prior to analysis to achieve an approximately normal distribution. All p values were two-tailed and p < 0.05 was considered to be significant. All statistical analyses were performed using the SPSS software (version 16.0; SPSS, Chicago, Ill., USA).

## Results

### Demographics of the Subjects

The total number of samples for proteomic analysis was 30, which consisted of the following: group A, 10 samples from HCC patients infected with HBV with BCP double mutations; group B, 10 samples from asymptomatic HBsAg carriers with BCP double mutations, and group C, 10 samples from HBsAg-negative individuals (table 1). The total number of samples for the validation of the significantly differentially expressed protein was 167, including 30 from HCC patients infected with HBV with BCP double mutations, 44 from asymptomatic HBsAg carriers infected with HBV with BCP double mutations, 47 from asymptomatic HBsAg carriers infected with HBV with wild-type BCP and 46 HBsAg-negative individuals (table 2).

### Identification of Differentially Expressed Proteins

**Fig. 1:**
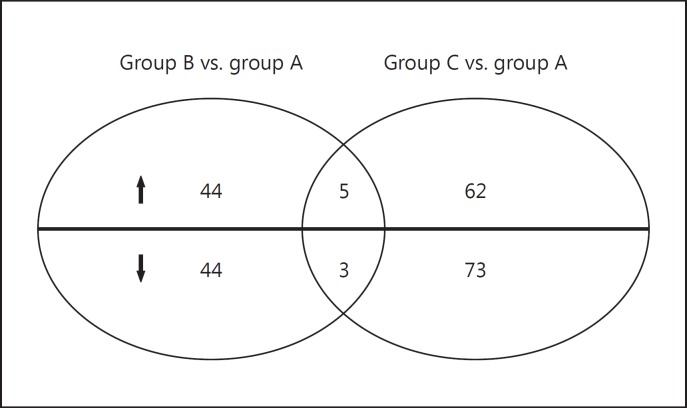
The numbers of upregulated (↑) and downregulated (↓) proteins. The number of upregulated proteins was 44 + 5 in group A versus B and 62 + 5 in group A versus C. The number of downregulated proteins was 44 + 3 in group A versus B and 73 + 3 in group A versus C. The numbers of upregulated and downregulated proteins in both group A versus group B and group A versus group C were 5 and 3, respectively.

The total number of proteins identified by iTRAQ was 1,125, of which 239 showed statistically significant differences in their expression when the relative protein concentrations of group A and group B, or group A and group C, were compared (fig. 1). The number of upregulated proteins in both group A versus group B and group A versus group C was 5, and that of the downregulated proteins in both group A versus group B and group A versus group C was 3 (table 3.).

### Validation of the iTRAQ Ratio by ELISA

The protein which showed the most significant correlation with liver diseases and infection was validated by ELISA. In this study, DLD was selected for the validation. In the results from iTRAQ, the relative concentrations of DLD in HCC cases with BCP double mutations, asymptomatic HBsAg carriers with BCP double mutations and HBsAg-negative individuals were 10, 12.69 and 14.43, respectively.

The concentrations of serum DLD in HCC cases with BCP double mutations, asymptomatic HBsAg carriers with BCP double mutations and HBsAg-negative individuals were 117.3 ± 33.8, 109.0 ± 34.5 and 205.1 ± 154.8 pg/ml, respectively. The difference in the concentrations of serum DLD between HCC cases with BCP double mutations and asymptomatic HBsAg carriers with BCP double mutations was not significant (t = 1.235, p = 0.221). However, the difference in the concentrations of serum DLD between the HBsAg-negative group and asymptomatic HBsAg carriers with BCP double mutations or the HBsAg-negative group and the HCC cases with BCP double mutations was significant (t = 4.105, p = 0.0001 and t = 3.714, p = 0.001, respectively), suggesting that the concentrations of serum DLD in those infected with HBV are lower.

In order to determine whether there is any difference in the concentrations of serum DLD between HBsAg carriers with BCP double mutations and those with the wild-type BCP, DLD from asymptomatic HBsAg carriers infected with HBV with the wild-type BCP were tested. The concentration of serum DLD in asymptomatic HBsAg carriers with the wild-type BCP was 167.6 ± 163.6 pg/ml. The differences between asymptomatic HBsAg carriers infected with HBV with the wild-type BCP and asymptomatic HBsAg carriers with BCP double mutations or HCC cases with BCP double mutations were significant (t = 2.376, p = 0.021, and t = 2.022, p = 0.048, respectively). However, the concentrations of serum DLD of asymptomatic carriers with the wild-type BCP were not significantly different from those of HBsAg-negative individuals (t = 1.129, p = 0.262). These results suggest that lower concentrations of serum DLD in individuals infected with HBV are attributable to BCP double mutations.

Multiple linear regression analysis was carried out to identify the factors that affect the concentrations of serum DLD. The independent variables included sex, age, BCP double mutations and ALT. HBeAg was not included because its correlation with BCP double mutations is more than 0.8. The results showed that ALT and BCP double mutations were associated with the concentrations of serum DLD (t = 2.463, p = 0.015, and t = 2.352, p = 0.020, respectively; table 4).

## Discussion

The major finding of this study is that HBV with BCP double mutations is associated with lower concentrations of serum DLD. The initial aim of the study was to identify serum proteins with differential concentrations between HCC patients and HBsAg asymptomatic carriers among individuals infected with HBV with BCP double mutations. We found that the relative concentrations of serum DLD differed significantly in the iTRAQ test between HCC patients and HBsAg asymptomatic carriers, and between HCC patients and HBsAg-negative individuals. However, only the difference between HCC patients and HBsAg-negative individuals could be confirmed by ELISA. Meanwhile, we found that the concentrations of serum DLD in those infected with HBV with BCP double mutations is significantly lower than individuals with the wild-type BCP. However, the difference was not seen between individuals with the wild-type BCP and those negative for HBsAg. Clearly, HBV with BCP double mutations are associated with lower concentrations of serum DLD.

There have been some studies aiming to identify serum biomarkers of HCC for early diagnosis using iTRAQ. These have compared proteins differentially expressed in serum between HCC and chronic hepatitis B patients [15,16]. In this study, we selected controls from those infected with HBV with BCP double mutations, representing individuals who have been confirmed to be at highest risk for the development of HCC [12].

DLD is the common flavoprotein component of the three mammalian α-keto acid dehydrogenase complexes, namely the pyruvate dehydrogenase complex, the α-ketoglutarate dehydrogenase complex and the branched-chain α-keto acid dehydrogenase complex. The DLD component is also present in the glycine cleavage system [17]. DLD has multiple roles in the energy metabolism and redox balance and is present in at least four multienzyme complexes [18]. A deficiency of DLD may lead to a range of presentations, mostly characterized by severe neurological impairment in early childhood with encephalopathy and seizures [19,20]. Liver injury is one of the results of DLD deficiency. Affected individuals frequently experience lifelong recurrent attacks of hepatopathy [21].

The association of viral infection with autoantibodies has been strongly suggested. Epstein-Barr virus, hepatitis B and C viruses, human immunodeficiency virus and human parvovirus B19 appear to be associated with autoantibodies more frequently than other viruses. Several studies have suggested that chronic HBV or HCV may act as a trigger factor for the development of autoimmune rheumatic diseases [22]. Autoantibodies are common in chronic HBV infection [23,24]. Autoantibody formation may occur in children with chronic HBV infection [25]. DLD has been found to be a major autoantigen in hepatitis C virus infection [26] and a target of autoantibodies in endometrial cancer [27].

BCP double mutations were first described in the core promoter of HBV from Japanese patients [7,28]. Subsequently, we and others found that the double mutations are associated with more severe liver diseases, including HCC [8,9,10,11]. The association between the double mutations and HCC has been confirmed by prospective studies [12,29,30]. However, the mechanisms of oncogenesis of BCP double mutations remain obscure.

The development of BCP double mutations is the result of immune selection [31] and the selection consolidates the persistence of HBV infection [32]. In this study, we found that individuals infected with HBV with BCP double mutations have lower concentrations of serum DLD than those with the wild-type BCP. It may be postulated that BCP double mutations may induce more autoantibody against DLD, resulting in lower concentrations of serum DLD. If so, it is not difficult to understand that BCP double mutations are associated with more severe liver diseases because the DLD deficiency may cause liver injury [21]. This may be one of the clues for the study of the mechanisms of oncogenesis of BCP double mutations.

Serum concentrations of ALT have been regarded as markers of liver injury, including a wide range of etiologies from viral hepatitis to fatty liver [33]. We found that the level of ALT is associated with the concentration of serum DLD. Sex and age were not found to be associated with the concentrations of serum DLD in this study. We did not determine the concentrations of DLD in acute and chronic hepatitis and liver cirrhosis but are planning to answer these questions in the future. Furthermore, a comparison of the concentrations of autoantibody against DLD between those infected with HBV with BCP double mutations and those with the wild-type BCP may help determine whether the mutations induce more autoantibodies against DLD.

## Disclosure Statement

No conflicts of interest exist for any of the authors.

## Figures and Tables

**Table 1:** Demographics of subjects for iTRAQ testing

Group	n	Male	Age, years	HBsAg (+)	HBeAg (+)	Anti-HBe (+)	ALT (≥40 IU/ml)
A: HCC-DM	10	10	48.8 ± 8.7	10	3	7	9
B: ASC-DM	10	10	48.5 ± 6.6	10	2	8	7
C: HBsAg (–)	10	10	47.9 ± 9.9	0	0	0	2

Total	30	30	48.4 ± 8.2	20	5	15	18

ASC = Asymptomatic HBsAg carriers; DM = double mutations.

**Table 2:** Demographics of subjects for validation testing

Group	n	Male	Age, years	HBsAg (+)	HBeAg (+)	ALT (≥40 IU/ml), %
HCC	30	28	52.1 ± 9.4	30	8	66.7 (20/30)
ASC-WT	46	27	50.8 ± 7.6	46	14	15.2 (7/46)
ASC-DM	45	24	43.8 ± 7.5	45	6	37.8 (17/45)
HBsAg (–)	46	34	30.5 ± 10.5	0	0	4.3 (2/46)

Total	167	113	43.6 ± 12.3	121	28	27.5 (46/167)

ASC = Asymptomatic HBsAg carriers; WT = wild-type; DM = double mutations.

**Table 3:** Up- and downregulated proteins in both group A versus group B and group A versus group C

Upregulated protein	iTRAQ ratio		Downregulated protein	iTRAQ ratio	
	A vs. B	A vs. C		A vs. B	A vs. C
Complement protein C4B			DLD	0.788	0.693
frameshift mutant			Isoform 2 of calcium-binding		
(fragment)	3.668	2.783	mitochondrial carrier protein Aralar2	0.62	0.595
Keratin, type II cytoskeletal			Isoform 2 of ribosome-releasing		
2 epidermal	2.223	2.067	factor 2, mitochondrial	0.542	0.582
Hemoglobin subunit beta	1.595	1.331			
Isoform M1 of pyruvate					
kinase PKM	1.494	1.382			
Eosinophil cationic protein	1.518	1.216			

**Table 4:** Multiple linear regression analysis for factors associated with the development of BCP double mutations

Variable	Unstandardized coefficients		Standardized Coefficients (beta)	t	Significance
	B	standard error			
(Constant)	2.057	0.127		16.197	0.000
Sex	0.020	0.041	0.045	0.484	0.629
Age	0.033	0.026	0.114	1.274	0.205
ALT	0.106	0.043	0.243	2.463	0.015
BCP	–0.096	0.041	–0.223	–2.352	0.020
